# Practical Hints upon Medical Examination for Life Assurance—V

**Published:** 1908-10-03

**Authors:** William P. S. Branson

**Affiliations:** Junior Medical Officer, Sun Life Office


### Life Assurance.

### Practical Hints upon Medical Examination for Life Assurance—V.

By WILLIAM P. S. BRANSON, M.D., M.R.C.P., Junior Medical Officer, Sun Life Office.

The Urine (*continued*).

The test for albumin, which consists in boiling the upper part of a column of urine and then adding a few drops of acetic acid, is rather easier to perform than the cold nitric-acid test as described in the fourth article of this series. It is also more delicate, and in this delicacy lies its chief drawback from the life assurance point of view; for it is capable of precipitating mucus as well as albumin, and this in a fashion which makes it impossible to distinguish how much of the haze produced is to be credited to each of the two constituents it may represent. Heller's test is therefore to be preferred as a general rule, especially when the examination is being conducted in a poor light.

We may pass now to a consideration of the vexed question, "What is the significance, with regard to longevity, of albuminuria apart from the presence of casts, blood, or any cardio-vascular indication of organic nephritis?" The frequency of this symptom is not quite sufficiently realised by the profession at large, for it is a fact that albumin, in sufficient quantity to be detected by the nitric-acid test, is found in some 10 per cent. of lives apparently healthy. It is pretty certain, therefore, that in a good many cases there is little baleful significance attaching to its presence. How, then, are we to distinguish the harmful from the harmless cases? The answer to this question, which is outlined in what follows, lays no claim to finality, but reflects the practice which experience has proved to be satisfactory for life-assurance work. Firstly, the age of the proposer is an important factor in judging the risk, for the potential dangers of such albuminuria as we are considering are likely to be deferred at least until the fiftieth year or thereabout. In consequence, albuminuria in a young man of twenty may be allowed far more leniency than a similar occurrence affecting a man of forty years and upward. At this latter age it is wise to assume an organic basis for the symptom, and to remember that intermittent albuminuria at this time of life often depends upon an excessive consumption of alcohol. In the second place, it is a rule of great importance that no life should be rejected on the score of albuminuria alone until a repetition of the test has proved that the anomaly is no mere accident of diet or exercise or some other of the little-known influences which govern the occasional appearance of albumin in the urine. The importance of this precaution is a business point. It is obvious enough that the unnecessary rejection of a life is a thing every office would wish to avoid, if only on the score of loss of good business. But there is a further item. In some people albumin is so haphazard in its appearances that their urine may to-day present a fifth of albumin, and to-morrow be absolutely normal. Summary rejection of such a person after one examination of the urine may be followed by his acceptance as a first-class life by another office on the succeeding day. An occurrence of this kind not only makes of the rejected man a damaging advertisement of the apparent rigours of the office which has refused him, but is disheartening to its agents, who feel they have been deprived, without due cause, of a well-earned commission. It is always well, therefore, to examine a faulty urine a second time, and to obtain a specimen of the water passed immediately upon rising from bed in the morning. Many of these functional cases of albuminuria are postural in origin.

Like albumin, sugar is one of the common surprises of life-assurance examinations. Like albumin also, it provides some difficult problems for solution. We may premise that diabetes, meaning thereby glycosuria with symptoms, but rarely comes the way of the assurance examiner, and, when it does, is easily disposed of, for cases presenting such symptoms are quite ineligible. Of simple glycosuria it may be said that it is common, and not by any means a necessary bar to life assurance. As regards the tests for sugar in the urine, Fehling's test enjoys a well-earned popularity. If it is freshly made it is efficient and trustworthy enough. Much has been written of the many substances, other than dextrose, which appear in the urine and are capable of reducing Fehling's solution. Without suggesting that such substances do not exist, the writer wishes to state emphatically that in a large majority of instances a urine which reduces Fehling's solution, even though the reduction be not complete, contains sugar in abnormal quantity. This conclusion is the result of numerous controlling experiments carried out by means of the phenylhydrazine test in actual assurance work. Now glycosuria may be as haphazard in its appearances as we have shown albuminuria to be. It is of equal moment in this case, therefore, that the urine should be examined more than once. Persistent glycosuria, even without symptoms, requires a recommendation of postponement. But a transient escape of sugar by the kidneys, say once in three examinations, may be purely dietetic in origin and negligible in connection with the life risk. This variety of glycosuria affects usually a male of full habit, and between forty and fifty-five years of age, not by any means necessarily intemperate, yet generous to himself in the matter of diet and little disposed to exercise. These risks assume a far more favourable complexion when the excess of appetite can be shown to be concerned chiefly with food and not with alcohol. In the latter case they are bad; in the former they may be recommended for acceptance as soon as the glycosuria has disappeared. Pyuria, except in the case of females, is always an indication for postponement. In females the possibilities of leucorrhœal contamination must be given weight. It is common to find in the urine of healthy males a few shreds of muco-pus, the relics of past gonorrhœa. In the absence of stricture and of all active trouble, these flakes may be neglected unless they are very plentiful.

